# Effect of Feedstock Composition on Physicochemical and Microbiological Properties of Compost from Tuber and Acidic Fruit Peel Residues

**DOI:** 10.1021/acsomega.6c02944

**Published:** 2026-09-07

**Authors:** Lizeth Natalia Bernal Peña, Fabian Bohorquez, Ellien Hernandez, Angie N. Morales, Wilson Caicedo, Diego Alejandro Rueda Cadavid

**Affiliations:** † Sustainable Development, Risk Management and Climate Change Research Group, 83534 Agrarian University of Colombia, Bogotá D.C. 11001, Colombia; ‡ Department of Mechanical Engineering, 28117 Federal University of Santa Catarina, Florianópolis, Santa Catarina 88040-970, Brazil; § NanoBio Research Group, Department of Chemical and Food Engineering, Federal University of Santa Catarina, Florianópolis, SC 88040-970, Brazil; ∥ Department of Chemical Engineering, Federal University of Santa Catarina, Florianópolis, Santa Catarina 88040-970, Brazil

## Abstract

The application of compost as a soil amendment can reduce dependence on chemical fertilizers, improve agricultural yields, and minimize the disposal of organic waste in landfills. However, the agronomic value of the resulting compost is strongly conditioned by the biochemical composition of the feedstock and the processing protocol. This study investigated how contrasting feedstocks affect thermal dynamics, physicochemical maturation, and microbiological safety indicators in two composting systems. Compost 1 (C1) was prepared from tuber peels of cassava (*Manihot esculenta*), potato (*Solanum tuberosum*), and carrot (*Daucus carota*) . In contrast, Compost 2 (C2) was prepared from acidic fruit peels of orange (*Citrus sinensis*), lemon (*Citrus limon*), pineapple (*Ananas comosus*), and passion fruit (*Passiflora edulis*). In both cases, sawdust and pruning residues were added as bulking agents. C1 reached a peak temperature of 69.1 °C and a final C/N ratio of 16.18, while C2 peaked at 57.5 °C and ended with a C/N ratio of 56.12. C2 presented higher moisture (74.01% vs 59.03% in C1) and higher available phosphorus (66.67 vs 45.83 ppm). Presumptive isolates compatible with *Salmonella*, *Escherichia coli*, and *Fusarium* were detected at process completion in one or both systems, identified at the genus level by selective media and morphology. Feedstock composition generated distinct composting trajectories that required differentiated management strategies; tuber-based substrates demanded alkalinity control to reduce nitrogen losses, whereas acidic fruit-peel mixtures required moisture adjustment and improved thermal management. The results demonstrate that physicochemical maturity and microbiological safety represent complementary but independent dimensions of compost quality, highlighting the need for direct microbiological verification even when conventional maturity indicators are achieved.

## Graphical abstract

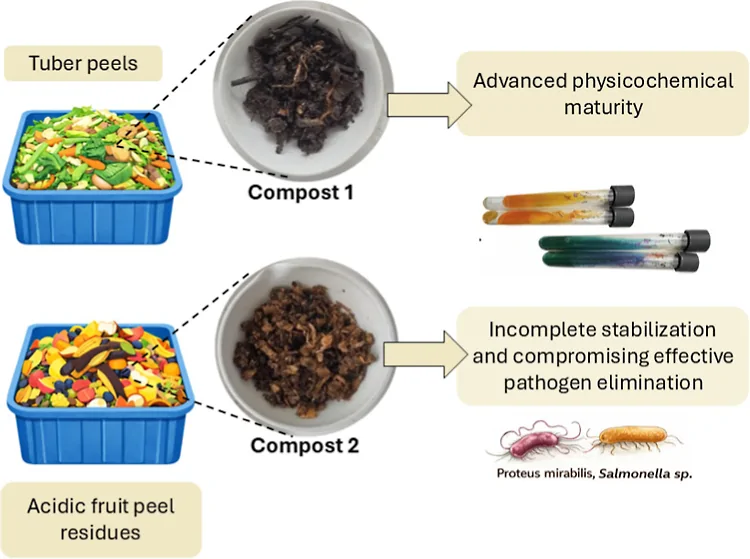

## 1 Introduction

Composting is one of the oldest and most effective strategies for managing organic waste. It transforms residual materials into stable, nutrient-rich amendments that improve soil fertility and support sustainable agriculture.^1^ Urban population growth and the expansion of agrifood systems have sharply increased organic waste generation worldwide. This trend has overloaded solid waste infrastructure and the ecosystems that receive the residues.^2^ Composting recovers organic waste, reduces greenhouse gas emissions associated with landfilling, and produces an amendment that restores degraded soils. Composting is especially useful in developing countries, where organic matter dominates the composition of municipal solid waste.^3^

Tuber peels from cassava, potato, and carrot, together with acidic fruit peels from orange (*Citrus sinensis*), lemon (*Citrus limon*), pineapple (*Ananas comosus*), and passion fruit (*Passiflora edulis*), are abundant organic fractions generated by households, restaurants, and markets.^4^ Their biochemical compositions differ, and these differences can drive distinct composting trajectories and microbial community dynamics.^5^ Tuber residues are rich in starch, labile carbohydrates, and nitrogenous compounds, favoring rapid growth of bacteria such as *Bacillus*, *Pseudomonas*, and *Enterobacteriaceae*.^6,7^ Acidic fruit peels, by contrast, contain organic acids and bioactive compounds that may inhibit microbial activity during early composting. For instance, Bromelain and Passion-fruit phenolics act mainly through proteolytic and antioxidant mechanisms, with limited direct antimicrobial activity under composting conditions.^8,9^ Together, these metabolites may reduce thermophilic development and compromise sanitization.^10^ Limonene typically accounts for about 90–95% of the essential oil of *Citrus* spp. and has documented membrane-disrupting activity against thermophilic bacteria and fungi.^11,12^ These contrasts argue against a universal composting protocol and call for feedstock-specific management, especially in developing countries.^13^

Colombia faces a growing challenge in organic waste management, as national solid waste generation rose by 36% between 2012 and 2022, reaching over 24 million tonnes nationwide.^14^ According to the National Planning Department, nearly 34% of the food produced in the country is wasted. The most affected groups are fruits and vegetables (62%), roots and tubers (25%), and cereals, meats, oilseeds, legumes, and dairy products (13%).^15,16^ In 2017, Bogotá generated about 7000 tonnes of solid waste per day, all of which was sent to landfill. The waste was dominated by organic matter (51.32%), followed by plastics (16.88%), cellulosic materials (13.67%), textiles (4.54%), glass (3.67%), and 9.93% comprising wood, metals, hazardous materials, and other residues. Most of the organic fraction comes from the food industry, restaurants, supermarkets, and households, with fruit, vegetable, and tuber residues as the predominant components.^17^

Inadequate disposal of this organic fraction has clear environmental and public health impacts. These include saturation of landfill capacity, contamination of surface waters by untreated leachates, odor emissions, and PM10 particulate matter exceeding permissible limits.^18–20^ Composting offers a sustainable response to these problems. It can be applied in both open and closed systems and reduces emissions of volatile compounds and odor-generating gases.^3,18^ It also converts organic waste into amendments that improve soil structure, water retention, aeration, nutrient availability, and microbial activity, while lowering dependence on synthetic fertilizers.^21^

Organic waste can be converted into valuable products through several biological treatment routes, including aerobic composting, anaerobic digestion, and vermicomposting, each characterized by distinct operational conditions and microbiological dynamics.^22,23^ Aerobic thermophilic composting is the most accessible technology for small-scale household and market-residue contexts in regions and is the focus of the present study. Composting has been studied across a wide range of feedstocks and operational configurations. The most frequently investigated substrates include municipal solid waste, kitchen and market residues, animal manures, sewage sludge, agro-industrial by-products such as fruit processing waste, coffee pulp, and sugarcane bagasse, and lignocellulosic materials such as rice straw, wood chips, and sawdust.^24^ System configurations range from open windrow and turned-pile systems to forced-aeration static piles and in-vessel reactors. In-vessel systems generally offer better thermal control and shorter retention times. Windrow systems typically need longer processing periods and adequate turning frequency to ensure effective sanitization.^24,25^ In Colombia and Latin America more broadly, small-scale semi-closed or open-air systems predominate. The comparative effects of locally abundant yet biochemically contrasting feedstocks, such as Andean tuber residues and acidic fruit peels, on composting dynamics remain underexplored.

Early composting studies identified thermophilic temperature as the main sanitization mechanism. Recent work has shown, however, that pathogens such as *Salmonella* spp., *Escherichia coli*, and *Fusarium* spp. can survive thermophilic conditions when heat distribution within the pile is uneven or exposure times are too short.^24,25^ Sánchez-Monedero et al. reported that substrate composition directly influences pH dynamics, C/N ratio, and nitrogen losses through volatilization. They also reported that bioactive compounds in citrus residues, including limonene and flavonoids, selectively inhibit thermophilic microbial communities, which can limit the attainment of sanitization temperatures.^26^ Bernal et al. later showed that substrate-driven metabolic imbalances affect both physicochemical maturity and microbiological safety.^27^ Hargreaves et al. and Cerda et al. confirmed that biochemically contrasting substrates, including citrus residues and starch-rich materials, require feedstock-specific management to ensure both maturity and microbiological safety.^25,28^ Despite these advances, the comparative effects of Andean tuber residues and fruit peels on composting dynamics in semi-urban contexts in developing countries have received limited attention.

Hence, comparatively few studies have examined composting systems representative of tropical high-altitude agricultural regions, and practical interpretation continues to rely predominantly on evidence generated under temperate conditions.^29^ Existing composting literature on these feedstocks remains fragmented and largely single-substrate. Starch-rich tuber peels such as cassava have been composted and characterized in isolation, primarily as a phytotoxicity and soil-amendment question,^30^ while citrus and other acidic fruit-peel residues have been studied as a separate stream, focused on pH correction with alkaline amendments and on the inhibitory role of essential-oil compounds such as limonene during the thermophilic phase.^31,32^ To our knowledge, no previous study has placed a starch-dominated, nitrogen-rich substrate and an organic-acid-dominated, moisture-retentive substrate side by side under the same operational conditions, specifically, Andean tuber peels and acidic fruit peels under high-altitude, semi-closed conditions representative of Colombian small-scale operations, which precludes any direct, controlled comparison of their thermal, physicochemical, and microbiological trajectories against the technical criteria of NTC 5167.

Furthermore, the relationship between physicochemical maturity and microbiological safety is often addressed indirectly; most reports treat thermal exposure as a sufficient indicator of sanitization and infer pathogen elimination from time–temperature profiles rather than through direct microbiological verification. Recent metagenomic evidence indicates that pathogens with low optimal growth temperatures can persist in nominally mature compost through heat-shock-protein-mediated thermotolerance, independently of conventional maturity indicators, a limitation particularly relevant in rural tropical settings and small-scale operations, where access to laboratory equipment or analytical resources is frequently unavailable.^29,33^ The present study addresses these complementary gaps within a single experimental framework by comparing two biochemically contrasting feedstocks while jointly evaluating physicochemical maturity and microbiological safety. This integrated approach provides additional evidence for understanding how feedstock composition influences compost maturation and microbiological quality under tropical high-altitude composting conditions.

## 2 Materials and Methods

### 2.1 Study Area

The composting trials were conducted at the Agrarian University Foundation of Colombia (UNIAGRARIA), located in the northern zone of Bogotá, Colombia (4° 45′ 15″ N, 74° 03′ 11″ W). The site has a mean annual temperature of 18 °C, with daytime temperatures ranging from 18 to 20 °C and nighttime temperatures dropping to as low as 6 °C. Relative humidity fluctuates between 75 and 80% annually. The composting process was initiated in March 2024 and monitored systematically every 3 days over 80 days. This interval was selected as the study period because, within this timeframe, the temperature progressively stabilized near ambient conditions, indicating that the compost had reached a stable, mature state.

### 2.2 Feedstock and Composting Process

Two compost mixtures, Compost 1 (C1) and Compost 2 (C2), were prepared in semi-closed, uncovered polyethylene containers placed outdoors, with intermittent aeration achieved through weekly manual turning. Each container had a capacity of 3000 L and dimensions of ≈53 × 110 × 102 cm (width × length × height). The total fresh-weight charged to each pile was approximately 120 kg, distributed as 72 kg of fresh peel residues (60% w/w), 36 kg of dried pruning leaves (30% w/w) and 12 kg of sawdust (10% w/w) (Figure 1). The bulking fraction (dried leaves + sawdust) therefore represented 40% w/w of the total pile mass, consistent with the recommended bulking ratio for high-moisture food-waste composts.^34^ The comparatively low sawdust contribution is acknowledged as a methodological choice, constrained by the locally available structuring materials.

**Figure 1:**
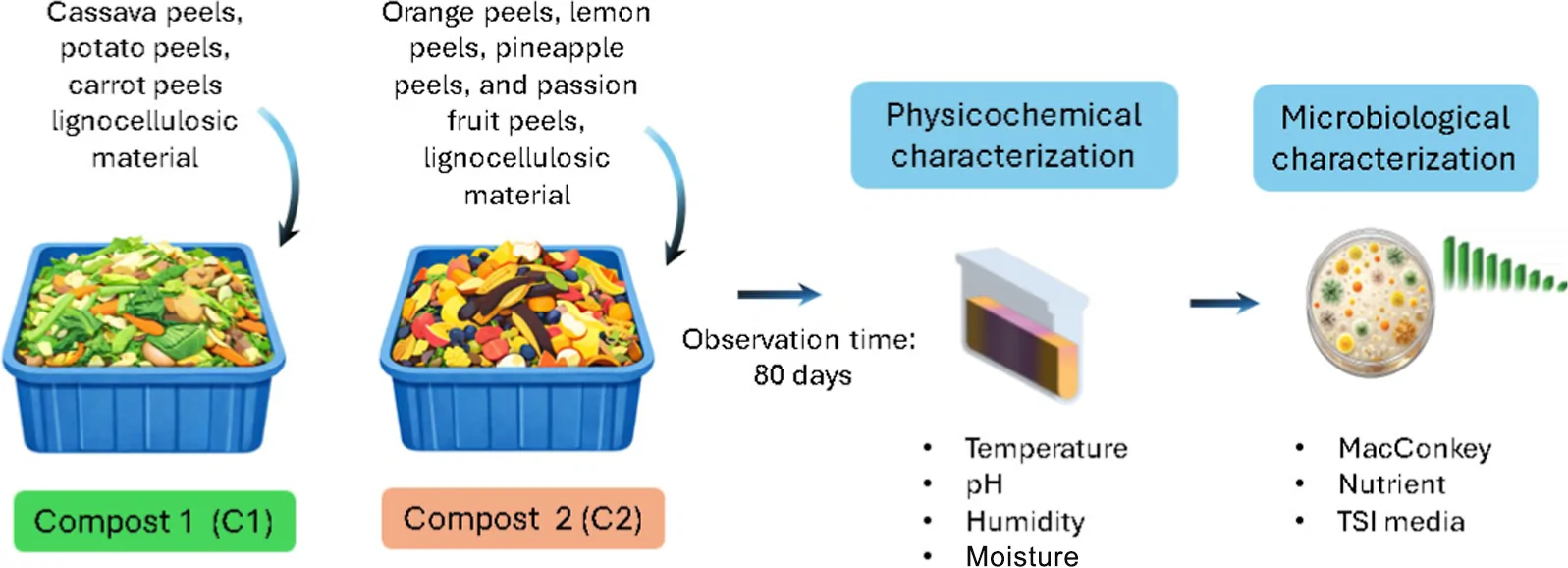
Schematic representation of the composting methodology. Two composting systems with different feedstock compositions (C1 and C2) were monitored over 80 days, followed by physicochemical (temperature, pH, humidity, and moisture) and microbiological (MacConkey agar, nutrient agar, and triple sugar iron (TSI) medium) characterization.

C1 consisted of tuber peels, namely cassava (*Manihot esculenta*), potato (*Solanum tuberosum*), and carrot (*Daucus carota*), collected as post-cooking residues from kitchens and markets near UNIAGRARIA. C2 consisted of fruit peels, namely orange (*Citrus sinensis*, Rutaceae), lemon (*Citrus limon*, Rutaceae), pineapple (*A. comosus*, Bromeliaceae) and passion fruit (*P. edulis*, Passifloraceae), collected from the same sources. Within the residue fraction, the peel types were combined in nominal target proportions (approximately one-third of each peel for C1 and one-quarter of each peel for C2). Both mixtures were combined with the same lignocellulosic bulking material, consisting of dried pruning leaves (30% w/w) and sawdust (10% w/w) of the total pile on a fresh-weight basis. Because direct elemental measurement of the freshly assembled mixtures was not performed at day 0, the initial carbon, nitrogen and C/N ratio of each pile were estimated from published feedstock-specific composition on a dry-mass basis, weighted by the fresh mass and dry-matter fraction of each component using the standard mixture formulation of Rynk et al.^35^

$$\frac{C}{N}=\{\left[\%\;C_{a}\times a\times \left(1-m_{a}\right)\right]+\left[\%{\;C}_{b}\times b\times \left(1-m_{b}\right)\right]+\left[\%{\;C}_{c}\times c\times \left(1-m_{c}\right)\right]+...\}/\{\left[\%{\;N}_{a}\times a\times \left(1-m_{a}\right)\right]+\left[\%{\;N}_{b}\times b\times \left(1-m_{b}\right)\right]+\left[\%{\;N}_{c}\times c\times \left(1-m_{c}\right)\right]+...\}$$

where *a*, *b*, and *c* represent the total weight of each ingredient used in the mixture, *m*_*a*_, *m*_*b*_, and *m*_*c*_ correspond to the moisture content of ingredients *a*, *b*, and *c*, respectively, while % N_*a*_, % N_*b*_, and % N_*c*_ represent the nitrogen content of each ingredient on a dry weight basis. % C_*a*_, % C_*b*_ and % C_*c*_ correspond to the carbon content of each ingredient. Initial C, N, and C/N values were estimated from literature data and were not experimentally determined. The literature reference values applied to each component are summarized in Table 1.

**Table 1:** Literature Reference Values Used to Estimate the Theoretical Initial Composition for C1 and C2 Mixtures (Dry Basis), Where DM (%) Refers to Dry Matter Content, % C to Carbon Content, % N to Nitrogen Content, and C/N to the Carbon-to-Nitrogen Ratio

component (dry basis)	DM (%)	% C	% N	C/N	refs
cassava peel	2.4	44.3	1.4	30.1	^30^,^36–38^
potato peel	7.7	43.7	4.0	22.8	^39–42^
carrot peel	12.4	23.8	0.8	27.0	^35^,^43^,^44^
orange peel	84.5	45.4	1.3	33.6	^45–48^
lemon peel	70.5	49.9	0.9	55.4	^43^,^49^,^50^
pineapple peel	88.5	49.3	3.1	15.9	^50^ and^51^
passion fruit peel	81.4	39.2	0.76	51.6	^35^,^52^
dried pruning leaves	38.0	49.0	0.9	47.0	^35^,^53^
sawdust	39.0	50.0	0.2	442	^35^

The markedly different initial theoretical C/N ratios likely contributed substantially to the divergent composting trajectories observed between C1 and C2. These literature-anchored baselines, although estimated rather than measured, provide the reference against which the final C/N values reported in Section 3.2 are interpreted. The degree of ripeness of the feedstocks influences the concentration of readily degradable substrates (simple sugars, labile carbohydrates), organic acids, bioactive compounds, and initial moisture content, thereby affecting composting kinetics and thermal dynamics. Feedstocks were assessed qualitatively at collection: tuber peels, oranges, and lemons at commercial maturity; and pineapple and passion fruit at full ripeness, based on outer skin coloration. Quantitative ripeness indices (°Brix, titratable acidity) were not measured, which is acknowledged as a limitation of feedstock characterization. Each composting treatment (C1, C2) was established as a single pile (one 3000 L container per treatment). Therefore, all statistics reported in this work reflect analytical replication (*n* = 3 measurements per sampling event) rather than independent batch replication.^54,56^

### 2.3 Physicochemical Measurements

Pile temperature and pH were monitored every 3 days throughout the 80 day observation period, providing a continuous time series record of the thermal and acid–base evolution of each system. The remaining physicochemical parameters, namely electrical conductivity, moisture content, ash content, total organic carbon, total nitrogen, C/N ratio, available phosphorus and exchangeable potassium, were determined at a single end-point sampling event at day 80, in analytical triplicate from a single composite sample prepared by homogenizing three sub-samples taken at three positions within each pile (approximately 20, 40, and 60 cm below the surface, corresponding to the upper, middle and lower zones). This sampling design was necessitated by the pilot-scale nature of the trial.

pH was measured on a 1:10 (w/v) suspension prepared by mixing 5 g of dry homogenized compost with 50 mL of deionized water, agitated for 30 min at 150 rpm on an orbital shaker, and allowed to settle for 30 min before reading, following the convention adopted in standard compost-characterization protocols Thompson et al.,^57^ and Bernal et al.^27^ pH data were taken with a digital pH meter (Hanna HI98103, accuracy of 0.2 pH units) calibrated before each session with NIST-traceable certified buffer solutions at pH 4.01, 7.01, and 10.01 (Hanna HI70004/HI70007/HI70010). Electrical conductivity was measured at the end-point on the same 1:10 (w/v) suspension using a conductivity meter (Hanna HI98129) calibrated with a NIST-traceable certified standard of 1413 μS/cm at 25 °C. Pile temperature was recorded with a digital thermometer (WMETERS TP101, accuracy 0.1 °C) inserted into the geometric center of each pile at 72 h intervals. Center-point data readings miss the thermal heterogeneity of the pile, since wall and surface cold spots commonly occur in small-scale reactors; the peak values reported here are therefore an upper bound on bulk exposure and do not certify sanitization (see Section 3.3).

Moisture and ash content were determined gravimetrically on the day 80 composite sample following NTC 5167. Total organic carbon was determined by loss-on-ignition at 550 °C for 4 h on oven-dried material, with organic carbon estimated as organic matter divided by the Van Bemmelen factor.^58^ The resulting C/N values should therefore be interpreted as comparative within this study rather than as absolute determinations. Total nitrogen was determined by Kjeldahl digestion in concentrated H_2_SO_4_ with a K_2_SO_4_/CuSO_4_ catalyst at 420 °C, followed by distillation and titration with 0.1 N HCl, according to AOAC Official Method 984.13. Available phosphorus was extracted by the Bray-2 method,^59^ and quantified colorimetrically by the molybdenum-blue method. Exchangeable potassium was determined by 1 M ammonium acetate extraction at pH 7.0, following the ICA Colombia protocol, consistent with the Soil Science Society of America Methods of Soil Analysis, Part 3.^60^ Calibration curves for phosphorus and potassium were constructed with certified analytical-grade KH_2_PO_4_ and KCl standards. The C/N ratio was calculated as the quotient of total organic carbon and total nitrogen. Analytical traceability rests on the NIST-certified pH and conductivity standards listed above and on the analytical-grade KH_2_PO_4_ and KCl.

### 2.4 Microbiological Analysis

Microbiological characterization was performed at the end of the composting period (day 80) on a composite sample obtained by homogenizing three positional sub-samples per pile, following the APHA Standard Methods for the Examination of Water and Wastewater^61^ and ISO 7218:2007.^62^ For each treatment, 10 g of compost was suspended in 90 mL of sterile distilled water, manually agitated for 10 min to ensure homogeneity, serially diluted, and 0.1 mL aliquots of the 10^–3^, 10^–4^ and 10^–5^ dilutions were surface-spread on the corresponding selective media. Total heterotrophic bacteria were enumerated on Nutrient Agar, Enterobacteriaceae and presumptive *E. coli* on MacConkey Agar (35 °C ± 0.3 °C, 24 h), thermotolerant coliforms on MacConkey Agar at 44.5 °C ± 0.2 °C following ISO 4832:2006, and fungi on Potato Dextrose Agar (PDA, room temperature, 8 d). Colony counts were taken only from plates within the 25–250 colony range per 0.1 mL plated, in compliance with ISO 7218:2007.^62^ Microbial loads were expressed as colony-forming units per gram of compost on a dry-weight basis (cfu·g^–1^ DW) using cfu·g^–1^ DW = (*N* colonies × dilution factor^–1^)/(*V* plated × dry-weight fraction), with *V* = 0.1 mL and dry-weight fractions of 0.41 (C1) and 0.26 (C2). Biochemical identification of presumptive isolates was performed using standard assays. For the triple sugar iron (TSI) test, each isolate was inoculated by stabbing the butt and streaking the slant and incubated at 37 °C for 24–48 h in duplicate. Reactions were read from the phenol red indicator as an acid (yellow) or alkaline (red) slant and butt. The Simmons Citrate test identified isolates able to use citrate as the sole carbon source, evidenced by alkalinization (blue in the bromothymol blue indicator), under the same incubation conditions. Microbial identification was based on selective culture media, colony morphology, Gram staining, and conventional biochemical assays rather than molecular methods. Therefore, the microorganisms reported throughout this study should be regarded as presumptive isolates. In addition, microbial loads were quantified as colony-forming units (cfu) instead of the most probable number (MPN) required by compost quality standards. Accordingly, the microbiological data should be interpreted as qualitative indicators of sanitary status rather than as direct evidence of compliance with regulatory microbiological criteria.

## 3 Results and Discussion

### 3.1 Temperature and pH of Stages C1 and C2

Temperature and pH trajectories for C1 and C2 are shown in Figure 2. Both systems started near ambient temperature (∼22 °C) and rapidly increased to approximately 41 °C within the first week, corresponding to the mesophilic phase, during which heterotrophic microorganisms actively degraded readily biodegradable organic substrates.^63^ Thereafter, distinct thermal profiles were observed. For C1, the thermophilic phase was initiated by a rapid temperature increase, reaching a maximum of 69.1 °C on day 10. This was followed by a temporary decline below 40 °C between days 13 and 19, after which thermophilic temperatures (>40 °C) were re-established from approximately day 22 to day 43, suggesting a renewed period of microbial activity.

**Figure 2:**
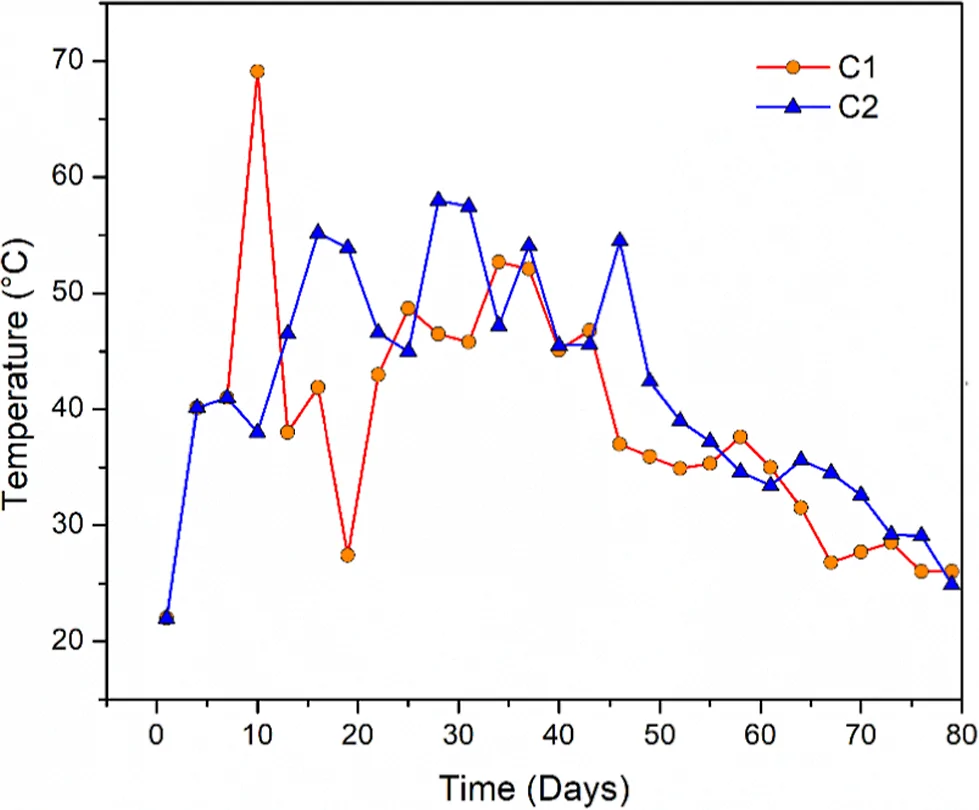
Temperature evolution of the C1 and C2 composting processes over an 80 day composting period.

In contrast, C2 exhibited a more gradual temperature increase, reaching a maximum of 58 °C on day 28 and maintaining thermophilic temperatures more continuously from approximately day 13 to day 49, remaining within the temperature range considered favorable for efficient organic matter degradation without adversely affecting microbial diversity.^64^ Subsequently, both systems entered the cooling phase, characterized by a gradual decrease in temperature. C2 maintained slightly higher temperatures than C1 during the early cooling stage, including a secondary increase to approximately 54.5 °C around day 46, whereas C1 showed a more continuous decline after day 43. Finally, during the maturation phase, both treatments gradually approached near-ambient temperatures (∼25–30 °C), with no further significant thermal peaks, indicating reduced microbial activity and progressive compost stabilization. pH variations were subtler than temperature changes but still differed between systems (Figure 3). Both piles rose from 6.2 to ∼9.5 during the mesophilic phase. C1 stayed in the 9.0–9.9 range across the remaining phases, while C2 fluctuated more widely (8.1–9.4) and stabilized at slightly lower values by maturation.

**Figure 3:**
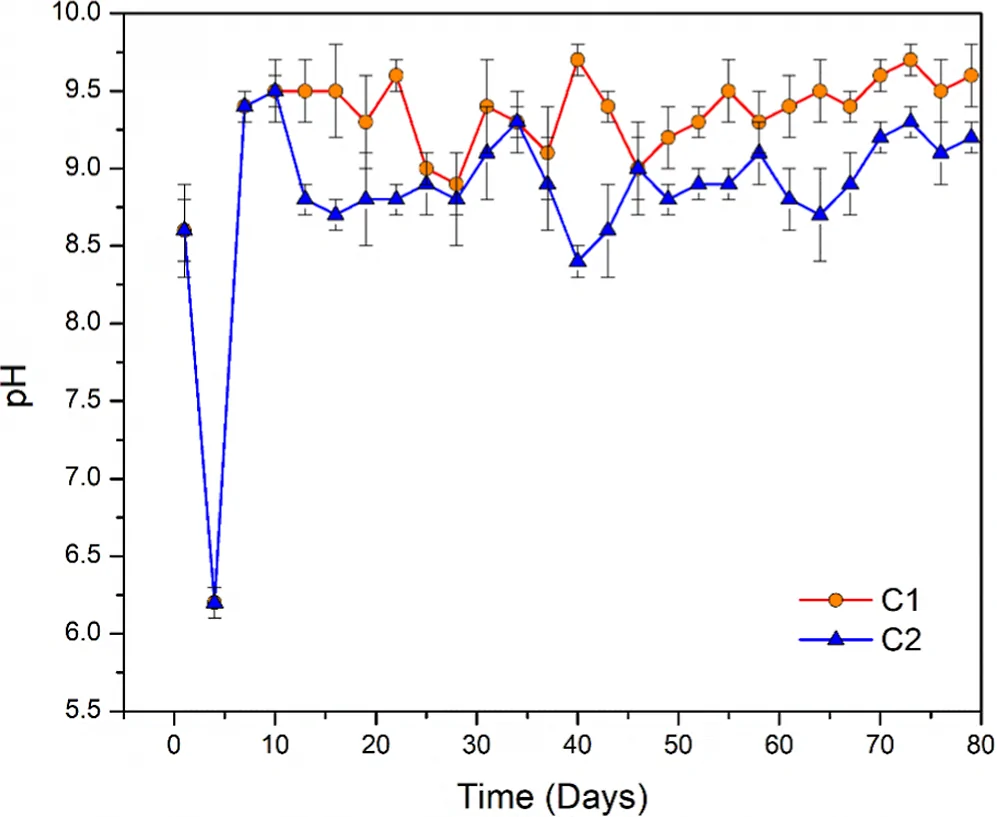
Evolution of pH values during the C1 and C2 composting processes over an 80 day composting period.

C1 remained consistently more alkaline, while C2 stabilized earlier and at high pH values. The overall pattern, alkalinization during the active and thermophilic phases followed by stabilization toward maturation, is consistent with previous reports in the literature.^65^ The persistent C1 pH of 9.0–9.9 likely constrained microbial succession and would have promoted NH_3_ volatilization, since ammonia emissions rise sharply above pH 7.5–8.0 in nitrogen-rich systems.^66,67^ Across the monitoring period, C1 and C2 shared a similar overall pattern, with C1 reaching a higher peak temperature (69.1 °C vs. 57.5 °C) and declining more rapidly toward ambient values. This trajectory points to more rapid early degradation. An acidification event (pH ≈ 6.2 on day 4) was observed in both systems, consistent with the accumulation of organic acids during the early mesophilic phase reported in previous composting studies.^68,69^ C2 exhibited a more moderate thermophilic phase and greater pH stability (max. 9.4), patterns that are consistent with slower mineralization and lower NH_3_ losses.^34^ Given that each treatment was represented by a single composting pile, comparisons among treatments are reported as qualitative time-series trends rather than as statistically validated differences.

### 3.2 Physicochemical Analysis

The physicochemical results are presented in Table 2, which summarizes properties of C1 and C2 composts at process completion. C1 showed higher electrical conductivity (2703 μS/cm) than C2 (1964 μS/cm) within the analysed composite samples. Electrical conductivity reflects the cumulative concentration of soluble ionic species, including NH^4+^ and NO^3–^ released during nitrogen mineralization, exchangeable K^+^, Na^+^, Ca^2+^ and Mg^2+^, Cl^–^ and SO_4_^2–^, and soluble organic anions from organic-matter mineralization.^25,27,63^ C1 lies within the 1500–4000 μS/cm range typical of nitrogen-rich food-waste co-composts during active decomposition.^63^ Still, it exceeds the 2000 μS/cm threshold sometimes associated with potential salinity stress in sensitive crops,^28^ a consideration when defining the agronomic dose for the C1 product.

**Table 2:** Summary of Physicochemical Parameters for C1 and C2 at Process Completion^a^ ^,^ ^b^

parameter	C1 (mean ± SD)	C2 (mean ± SD)	comparison
conductivity (μS/cm)	2703 ± 617.52	1964 ± 232.95	C1 > C2
moisture (%)	59.03 ± 3.98	74.01 ± 2.06	C2 > C1
ashes (%)	5.31 ± 1.32	0.95 ± 0.19	C1 > C2
density (g/mL)	0.30 ± 0.07	0.29 ± 0.04	C1 = C2
C/N experimental	16.18 ± 3.92	56.12 ± 11.23	C2 > C1
phosphorus (ppm)	45.83 ± 7.22	66.67 ± 14.43	C2 > C1
potassium (ppm)	76.67 ± 5.77	76.67 ± 5.77	no difference

a Mean ± SD computed from *n* = 3 analytical replicates of a single end-point composite sample per treatment. The “comparison” column reports descriptive analytical variability between the two composts.

b C1: compost 1, C2: compost 2.

Within the analyzed composite samples, moisture was higher in C2 (74.01%) than in C1 (59.03%) (Table 2). The C2 value exceeds the 40–65% range recommended for aerobic composting and is comparable to the 65–75% range reported by Petric et al. for piles in which restricted aeration limited thermogenesis. Excessive moisture in C2 likely impaired oxygen diffusion, promoting the formation of anaerobic pockets and associated methane production.^70^

Ash content was higher in C1 (5.31%) than in C2 (0.95%) among the analyzed composite samples, indicating more advanced organic-matter mineralization in C1 and a more mature, stable product. The lower ash content in C2 suggests that decomposition was still in progress and would require additional time to reach a comparable level of maturity.^71^ Bulk density was similar between systems (Table 2) but fell below the ideal range for mature compost. The slightly higher value in C1 is consistent with a more compact, further-decomposed matrix.^72^

The experimental C/N ratios (Table 2) diverged sharply from the theoretical initial values in Table 1. C1 evolved from an estimated initial C/N ≈ 22.23 (Section 2.2) to a final value of 16.18 ± 3.92, placing the product within the maturity range conventionally accepted for stabilized composts (C/N < 20).^27^ C2 evolved from an estimated initial C/N ≈ 64.44 to a final value of 56.12 ± 11.23, indicating only a limited reduction in the C/N ratio over the 80 days. Despite this decrease, the final value remained far above the maturity range typically reported for stabilized composts, suggesting restricted nitrogen conservation and incomplete organic matter stabilization. These interpretations should be considered because the initial C/N values represent theoretical estimates rather than direct measurements of the compost mixtures. Hence, although alkaline pH likely promoted NH_3_ volatilization in C1, carbon mineralization and CO_2_ losses apparently exceeded nitrogen losses, resulting in a net decrease of the C/N ratio,^9,10^ and restricted aerobic mineralization at 74% moisture.

Although the initial C/N values were estimated rather than measured, the divergence between C1 and C2 over the 80 day window is too large to be explained by baseline uncertainty alone. Only C1 progressed toward stabilization, whereas C2 retained a persistently elevated C/N ratio throughout the process. This contrast likely represents an important driver and should be confirmed in replicated trials with direct characterization of the feedstock. The final C/N of C1 (16.18) falls within the 10–20 range broadly accepted as indicative of mature compost,^27^ and is consistent with values reported for food-waste composts.^73^ The final C/N of C2 (56.12) substantially exceeds the typical 18–35 range reported for citrus and food- waste composts amended with manures or co- substrates^11,21^ and approaches values reported for unbalanced, lignocellulosic- rich mixtures with high carbon load.^67^ The C2 moisture (74%) is comparable to literature values in which pile sub-aeration was identified as a limiting factor (65–75%).^70^ The electrical conductivity of C1 (2703 μS/cm) is within the 1500–4000 μS/cm range reported for nitrogen- rich food- waste co- composts during active decomposition.^63^

Nutrient analysis revealed that C2 had a higher phosphorus concentration (66.67 ppm) than C1 (45.83 ppm), making it a better candidate for phosphorus-deficient soils. Phosphorus is essential for root development, root architecture, and nutrient absorption efficiency, and its availability can regulate root morphology and overall plant growth.^74^ The higher total phosphorus concentration in C2 may therefore benefit crops established under low-fertility conditions. It should be noted, however, that more than 50% of mineral phosphorus in compost may be associated with poorly exchangeable, slow-release forms such as calcium phosphates. Further compost maturation promotes mineralization and microbial activity, progressively increasing plant-available phosphorus fractions over time.^75^ Both composts presented similar potassium levels of approximately 76 ppm, an element essential for enzymatic activation and key physiological processes in plant growth. The nutritional composition of compost is largely determined by the type of biowaste used, the composting method applied (industrial or small-scale), the use of enrichment treatments, and the degree of maturity achieved.^74,76^

The C/N ratio and ash content tracked mineralization progress in C1 but cover only part of compost maturity. Complementary indicators of phytotoxicity, microbial stability, and humification, such as the Zucconi germination index,^77^ cumulative CO_2_ respiration,^78^ the humic-to-fulvic acid ratio,^79^ and the Solvita Compost Maturity Test, were beyond the scope of this work. Given the alkaline pH of C1 (9.3–9.9) and the associated risk of free-ammonia phytotoxicity,^51^ a Zucconi bioassay on *Lepidium sativum* or *Lactuca sativa*, with a target germination index of at least 80%, is recommended before any agricultural use of C1.

### 3.3 Microbiological Analysis

Microbiological analysis of C1 and C2 revealed distinct microbial profiles, including microorganisms of sanitary concern (Tables 3 and 4). On MacConkey agar at the 10^–3^ dilution, 608 colonies were counted in C1 compared with only 7 in C2, indicating a substantially higher Enterobacteriaceae load in C1. Colony counts are presented as raw values at the corresponding dilutions and were converted to cfu·g^–1^ DW using dry-weight fractions of 0.41 for C1 and 0.26 for C2, calculated from the Day 80 moisture contents. Microbial identification was performed based on colony morphology, Gram staining, selective culture media, and conventional biochemical assays (TSI and Simmons citrate). Because no molecular confirmation (e.g., PCR or sequencing) was performed, the microorganisms identified in this study are reported as presumptive isolates. Consequently, the microbiological findings should be interpreted as indicators of the sanitary quality of the composts rather than as definitive evidence of pathogen occurrence.

**Table 3:** Bacteria and Fungi Identified in C1 and C2 Using MacConkey, Nutrient, and PDA Media

		C1			C2		
		cfu·g^–1^	name/staining	shape	cfu·g^–1^	name/staining	shape
bacteria	thermotolerant	<1 × 10^–5^	-		<1 × 10^–5^		
	MacConkey	608 × 10^–3^	-	-	7 × 10^–3^	-	-
		90 × 10^–4^	negative	cocci	1 × 10^–4^	-	-
	nutrient	3 × 10^–5^	positive	bacillary	<1 × 10^–5^	-	-
		520 × 10^–3^	positive	bacillary	416 × 10^–3^	-	-
		95 × 10^–4^	-	-	>200 × 10^–4^	-	-
		55 × 10^–5^	-	-	7 × 10^–5^	positive	bacillary
fungi	PDA	6 × 10^–4^	Fusarium spp.	elongated Bacillus with rounded edges	3 × 10^–4^	Cladosporium spp.	elongated Bacilli with rounded edges
		2 × 10^–5^	Fusarium spp.	elongated Bacillus with rounded edges	-	-	-

**Table 4:** Biochemical Confirmation Assays (TSI, Simmons Citrate) Support the Identification Reported in Table 3

			C1			C2	
	concentration		name/staining	shape		name/staining	shape
TSI	10^–3^	-	-	-	+	√Proteus mirabilis	-
						√Salmonella sp	
	10^–4^	+	√Escherichia coli		-	-	-
			√Klebsiella pneumoniae				
simmons citrate	10^–3^	-	-		-	-	-
	10^–4^	+	√Klebsiella pneumoniae		-	-	-
			√Salmonella spp.				
			√Pseudomonas aeruginosa				

On nutrient Agar, Gram-positive bacilli were detected in C1 at the 10^–3^ dilution, whereas in C2 they were observed only at the 10^–5^ dilution, suggesting a higher total microbial load in C1 (Table 4). Spore-forming Gram-positive bacteria, particularly of the genus *Bacillus*, are typically associated with the thermophilic phases of composting and contribute substantially to the degradation of recalcitrant organic compounds.^80,81^ It is important to note, however, that total heterotrophic bacterial counts alone do not guarantee the absence of pathogens or the presence of complete microbiological maturity, as population fluctuations can occur even after the thermophilic phase.^52^ Therefore, although the higher bacterial abundance observed in C1 suggests a more active microbial community at the end of the composting process, it should not be interpreted as an indicator of compost maturity or microbiological safety.

The TSI biochemical assay provided biochemical characteristics consistent with presumptive *E. coli*- and *Klebsiella pneumoniae*-like isolates in C1 at the 10^–4^ dilution. Because identification was based exclusively on culture characteristics and biochemical assays, these microorganisms should be regarded as presumptive isolates rather than confirmed pathogens. Consequently, their detection should be interpreted as an indicator of potential microbiological risk rather than definitive evidence of pathogen occurrence.^82^ The detection of *E. coli* is a sanitary concern because it indicates fecal contamination and the potential presence of other enteric pathogens. The absence of thermotolerant colony-forming units at 44.5 °C in both composts (Figure 2 and Table 3) points to a reduction of this thermal-tolerance indicator during the process, although the 72 h centre-point monitoring used here cannot quantify it. Literature benchmarks for adequate thermophilic composting include approximately 4 log_10_ reduction of *E. coli* and final concentrations below 64 MPN·g^–1^ dry matter,^83^ with 55 °C sustained for 5 days in aerated systems sufficient for sanitization.^84^ The microbiological quality criteria established by NTC 5167 and the Test Methods for the Examination of Composting and Compost are expressed as most probable number (MPN) per unit dry mass, whereas the present study quantified cultivable microorganisms as colony-forming units (cfu). Consequently, direct demonstration of regulatory compliance is not possible. Nevertheless, the detection of presumptive *E. coli*-like isolates at the end of the composting process indicates that microbiological safety cannot be assumed solely from physicochemical maturity or thermophilic temperature profiles, highlighting the importance of direct microbiological evaluation.

C2 showed *Proteus*-like and *Salmonella*-like presumptive isolates at the 10^–3^ dilution, both compatible with microorganisms of sanitary concern. *Proteus*-like isolates have been recovered from composts prepared with manure and animal organic residues, and *Salmonella*, in particular, is a public health risk because of its pathogenicity.^85^ Reported decimal reduction times (*D*-values) for *Salmonella* spp. in compost matrix range from 3.6 to 18 min at 60 °C, depending on water activity and matrix protection,^84^ and inactivation typically requires temperatures of 50–65 °C maintained for several days.^7,84,85^ Therefore, the detection of presumptive *Salmonella*-like isolates should be interpreted qualitatively as evidence suggesting incomplete sanitization requiring further maturation and confirmatory molecular analyses. Then, comparisons with the regulatory standard should be regarded as qualitative rather than quantitative. The detection of presumptive isolates compatible with *E. coli*, *Salmonella*, and *Fusarium* in C1, despite reaching 69.1 °C, agrees with previous findings indicating that pathogen survival is the rule rather than the exception when time–temperature criteria are not met.^55,71^ The detection of *Cladosporium*-like rather than *Fusarium*-like isolates in C2 is consistent with fungal succession patterns previously reported for high-moisture compost recipes.^86^ These findings suggest that the thermophilic phase achieved in C2 was insufficient to ensure microbiological sanitization despite reaching temperatures commonly associated with pathogen inactivation, emphasizing that both temperature and exposure time are critical determinants of compost hygienization.

On Simmons Citrate Agar, presumptive isolates of *Pseudomonas*, *Klebsiella*, and *Salmonella* were detected in C1. *Pseudomonas* isolates have frequently been recovered from compost derived from agricultural by-products,^85^ and similar isolates have been documented during microbial succession in hydrocarbon-contaminated soil bioremediation, consistent with their capacity to degrade complex organic compounds.^24^ Members of this genus have frequently been reported during composting because of their ability to degrade complex organic compounds; however, species-level identification was beyond the scope of this study.

Mycological analysis revealed marked differences between the two composts (Table 4). C1 presented *Fusarium*-like isolates at the 10^–4^ and 10^–5^ dilutions on PDA medium, while C2 showed *Cladosporium*-like isolates at the 10^–4^ dilution. *Fusarium* is a common filamentous fungal genus in agricultural soils and has been reported in composts of diverse origins.^24,85^ Although members of this genus can participate in organic matter degradation during composting, several species are recognized phytopathogens capable of causing wilt and rot diseases in economically important crops. In contrast, the *Cladosporium*-like community in C2 is frequently associated with compost maturation phases and is considered an indicator of process progression. *Cladosporium* spp. is generally considered less phytopathogenic than *Fusarium* spp. in composting systems. A shift toward fungal communities dominated by *Cladosporium*-like rather than *Fusarium*-like genera is considered a positive indicator of microbiological progression. The general decline in fungal counts during composting is typically associated with the thermophilic phase and reduced availability of readily degradable substrates. It is considered a sign of progression toward product maturity.^87^

Compared with C2, compost C1 exhibited a greater abundance and diversity of cultivable microorganisms, particularly Gram-negative bacteria, heterotrophic bacteria, and fungi. These observations suggest a more metabolically active microbial community at the end of the composting process. However, the simultaneous detection of presumptive *E. coli*-, *Salmonella*-, and *Fusarium*-like isolates indicates that higher microbial activity should not be interpreted as evidence of microbiological safety. Likewise, although C2 presented lower cultivable bacterial counts and a fungal community dominated by *Cladosporium*-like isolates, the detection of presumptive *Salmonella*- and *Proteus*-like isolates also indicates evidence compatible with incomplete sanitization.^88^

The divergent behavior of C1 and C2 tracks the contrasting chemical composition of their initial feedstocks. The tuber-based mixture (C1) supported a more intense thermophilic phase but also maintained an alkaline pH (>9.5), which drove NH_3_ volatilization losses.^24,87^ In contrast, the acidic fruit-peel mixture (C2), combined with the high-water retention of sawdust, was associated with excessive moisture and lower peak temperatures that did not sustain 55 °C for the 5–7 days typically reported as necessary for *Salmonella* inactivation.^87^ The persistence of *Salmonella*-like, *E. coli*-like, and *Fusarium*-like presumptive isolates in C1, and of *Salmonella*-like and *Proteus*-like presumptive isolates in C2, is consistent with the hypothesis that feedstock composition influenced both thermal development and microbiological evolution.

These results define differentiated operational protocols for the two feedstocks. For tuber-based mixtures (C1-type), the priority is to contain the alkaline drift and the associated NH_3_ losses by increasing the proportion of lignocellulosic bulking material, incorporating alkalinity buffers such as biochar or gypsum to limit NH_3_ volatilization, replacing sawdust with lower water-retention bulking agents, and optimizing turning practices during the thermophilic phase. For acidic fruit-peel mixtures (C2-type), the priority is to overcome the excess moisture and the terpene-mediated suppression of thermophilic microbiota by pre-drying the peel fraction to reduce excessive moisture, replacing sawdust with coarser structuring agents such as straw or wood shavings, and improving aeration and heat retention to sustain an effective thermophilic phase. Both products require an extended maturation of 5–6 months with periodic microbiological monitoring, ideally with molecular confirmation, until microbiological monitoring confirms the absence of presumptive *Salmonella*-like isolates and compliance with regulatory criteria for *E. coli* using the analytical methodology required by the applicable standard.^89^

Within the analytical replicates of each treatment, C1 (tuber peels) reached a final C/N of 16.18 and a peak temperature of 69.1 °C, while C2 (acidic fruit peels) ended at C/N 56.12 with a peak of 57.5 °C and 74% moisture. Presumptive isolates compatible with *Salmonella*, *E. coli*, *Klebsiella*, *Pseudomonas*, *Proteus*, and *Fusarium* were detected in one or both products. The data suggest that both feedstock biochemistry and the markedly different initial C/N ratios contributed to the contrasting process outcomes. The exploratory design used here, based on a single pile per treatment, literature-derived initial C/N values, and 72 h centre-point thermal monitoring, calls for confirmation in a replicated study.

At industrial scale, larger pile mass, lower surface-to-volume ratio, lateral insulation, forced aeration, and scheduled turning sustain the 55 °C exposure for 5–15 days required for reliable *Salmonella* and *E. coli* inactivation. The persistence of presumptive pathogen-like isolates observed at the present 300 L scale may reflect the limited thermal performance of the experimental system rather than an intrinsic characteristic of the feedstocks. Collectively, these findings contribute to the current understanding of composting by showing that feedstock biochemical composition is associated with differences in both physicochemical development and microbiological quality. The starch-rich substrate promoted faster mineralization and greater thermal development, whereas the acidic fruit-peel substrate retained higher moisture and exhibited slower stabilization. Therefore, direct microbiological assessment should be considered a complementary criterion for evaluating compost quality, particularly when novel feedstocks with contrasting biochemical compositions are investigated. These findings also provide additional evidence to support the design and assessment of composting strategies for biochemically contrasting agricultural residues, particularly under tropical high-altitude conditions where experimental data remain limited.

## 4 Conclusions

We compared the composting of two contrasting feedstocks. Compost 1 (C1) combined tuber peels from cassava (*M. esculenta*), potato (*S. tuberosum*), and carrot (*D. carota*), whereas Compost 2 (C2) used acidic fruit peels from orange (*Citrus sinensis*), lemon (*Citrus limon*), pineapple (*A. comosus*), and passion fruit (*P. edulis*). Both treatments used the same lignocellulosic bulking material, consisting of dried pruning leaves and sawdust. The initial substrate composition produced clearly distinct thermal, physicochemical, and microbiological trajectories. Compared to C2, C1 reached a higher thermal peak (69.1 °C vs. 57.5 °C), achieved a substantially lower final C/N ratio (16.18 vs. 56.12), retained less moisture (59.0% vs. 74.0%), and retained less phosphorus. Driven by the high soluble-carbohydrate content of tuber peels, C1 reached physicochemical maturity with elevated ash content, whereas the alkaline pH above 9.5 and electrical conductivity of 2703 μS/cm indicate substantial nitrogen losses via NH_3_ volatilization. In C2, the combination of acidic organic matter and the high water-retention capacity of sawdust sustained elevated moisture throughout composting, which restricted aerobic mineralization; as a result, the C/N ratio decreased only from a theoretical initial value of 64.44–56.12, remaining well above the range associated with mature compost, despite higher phosphorus retention relative to C1.

Microbiological characterization detected presumptive *E. coli*-, *Salmonella*-, and *Fusarium*-like isolates in C1 and presumptive *Salmonella*- and *Proteus*-like isolates in C2, based on selective media and morphological characteristics without molecular confirmation. While both composts exhibited suitable progress toward physicochemical stabilization, the microbiological results indicate that additional maturation followed by microbiological evaluation using methods consistent with NTC 5167 (e.g., MPN) and molecular confirmation of presumptive isolates is recommended before direct agricultural use. Importantly, the higher phosphorus retention observed in C2 did not coincide with improved microbiological quality, highlighting that nutrient preservation and hygienic safety represent complementary but independent dimensions of compost quality that should be assessed separately.

The results demonstrate that physicochemical maturity and microbiological safety should be considered complementary but independent indicators of compost quality and, therefore, should be evaluated separately. The contrasting behaviour of the two feedstocks also suggests that compost management strategies may benefit from being adapted to the characteristics of each material. For tuber-based feedstocks, increasing the proportion of lignocellulosic bulking material and incorporating alkalinity-buffering amendments such as biochar or gypsum may help reduce excessive pH increases and ammonia losses. For acidic fruit-peel feedstocks, reducing the initial moisture content through pre-drying and using coarser bulking agents, such as straw or wood shavings, may improve aeration and composting performance. In both systems, extended curing combined with periodic microbiological monitoring would provide greater assurance of microbiological quality before agronomic use. Where microbiological criteria are not achieved after curing, additional hygienization measures or blending with more mature compost could also be considered.

Beyond the specific feedstocks investigated, this study contributes to composting science by demonstrating that feedstock biochemical composition influences not only decomposition efficiency but also the relationship between physicochemical stabilization and microbiological sanitization. These findings indicate that conventional maturity indicators alone are insufficient for assessing compost quality and support the incorporation of independent microbiological verification into evaluation protocols, particularly for heterogeneous food-waste composts. The proposed framework may therefore be applicable to a broader range of biochemically contrasting organic residues beyond those evaluated in the present work. Future research could further strengthen these findings by incorporating replicated composting piles to enable formal statistical evaluation of treatment effects, together with a broader set of compost maturity indicators, including the Zucconi germination index, phytotoxicity assays, cumulative CO_2_ respiration, and the humic-to-fulvic acid ratio. Direct characterization of the initial feedstock composition and molecular confirmation of presumptive microbial isolates would also provide a more comprehensive assessment of compost development and microbiological quality. These complementary approaches would contribute to a more robust evaluation of feedstock-specific composting strategies and support their safe and effective agronomic application.
